# The Progress of Induced Pluripotent Stem Cells as Models of Parkinson's Disease

**DOI:** 10.1155/2016/4126214

**Published:** 2016-01-06

**Authors:** Ji-feng Kang, Bei-sha Tang, Ji-feng Guo

**Affiliations:** ^1^Department of Neurology, Xiangya Hospital, Central South University, Changsha, Hunan 410008, China; ^2^State Key Laboratory of Medical Genetics, Changsha, Hunan 410008, China; ^3^Key Laboratory of Hunan Province in Neurodegenerative Disorders, Central South University, Changsha, Hunan 410008, China; ^4^Neurodegenerative Disorders Research Center, Central South University, Changsha, Hunan 410008, China

## Abstract

In recent years, induced pluripotent stem cells (iPSCs) were widely used for investigating the mechanisms of Parkinson's disease (PD). Somatic cells from patients with* SNCA* (*α*-synuclein),* LRRK2* (leucine-rich repeat kinase 2),* PINK1* (PTEN induced putative kinase 1),* Parkin* mutations, and at-risk individuals carrying* GBA* (*β*-glucocerebrosidase) mutations have been successfully induced to iPSCs and subsequently differentiated into dopaminergic (DA) neurons. Importantly, some PD-related cell phenotypes, including *α*-synuclein aggregation, mitophagy, damaged mitochondrial DNA, and mitochondrial dysfunction, have been described in these iPSCs models, which further investigated the pathogenesis of PD. In 2007, Takahashi et al. and Vodyanik et al. generated iPSCs from human somatic cells for the first time. Since then, patients derived iPSCs were applied for disease modeling, drug discovery and screening, autologous cell replacement therapy, and other biological applications. iPSC research has now become a hot topic in a wide range of fields. This review summarizes the recent progress of PD patients derived iPSC models in pathogenic mechanism investigation and potential clinical applications, especially their promising strategy in pharmacological study and DA neurons transplantation therapy. However, the challenges of iPSC transplantation still exist, and it has a long way to go before it can be used in clinical application.

## 1. Introduction

Induced pluripotent stem cells (iPSCs) are similar to human embryonic stem cells in their morphology, self-renewing capacity, and differentiation potential to any cell types. In 2007, Takahashi et al. and Vodyanik et al. induced adult human somatic cells into iPSCs by transferring a series of specific transcript factors (Oct4, Sox2, Klf4, c-Myc or Oct4, Sox2, Nanog, Lin28) [1, 2], which represented a new method to generate disease-specific pluripotent stem cells from the patients. From then on, the researches of iPSCs have come into a new milestone. Takahashi and Vodyanik generated iPSCs with retroviruses carrying transcription factors and oncogene c-Myc, which raise the risk of tumorigenicity and other side effects. Also, low efficiency of vector expression may limit the differentiation potential of the iPSCs. To overcome the potential safety risk and low efficiency, the protocols of iPSCs reprogramming and DA neuron generation have constantly been refined. Safer and more effective methods using nonintegrating vectors, synthetic modified mRNA, and small molecules (for SB431542, PD0325901, and Thiazovivin) instead of transgene integration have been attempted in many studies, which can directly activate the expression of transcription factors in iPSC generation through different ways. Furthermore, the course of generation can be controlled in a strict way and reduce the uncertainty risk of genetic alteration and transformation to the greatest extent. The new methods not only saved time but also improved reprogramming efficiency and safety [3–7], which provided an alternative strategy for disease modeling and clinical study.

## 2. Parkinson's Disease

Parkinson's disease (PD) is the second most common late-onset neurodegenerative disorder, clinically characterized by a series of motor symptoms such as bradykinesia, resting tremor, rigidity, and postural instability as a result of dopaminergic (DA) neuron degeneration in the substantia nigra pars compacta (SNpc), accompanied with sleep disorders, cognitive decline and other nonmotor symptoms [8]. PD is a complex disease with the combination of environmental exposures and genetic factors. About 10% of PD patients have a positive family history and a series of genes such as* SNCA*,* LRRK2*,* VPS35*,* Parkin*,* PINK1*,* DJ-1*,* PLA2G6*, and* ATP13A2* were cloned in familial PD patients [9]. The pathogenesis of PD is still elusive; mitochondria dysfunction, *α*-synuclein accumulation, mitophagy, and oxidative stress were thought to play an important role in the occurrence of PD [8].

Over the past hundred years, the therapeutic methods for PD were gradually and steadily developed. Dopaminergic therapy is the most effective symptomatic treatment drug for PD, while deep brain stimulation (DBS), entering into a new era of human neural-network modulation, improved the life quality of patients greatly [10]. Since the discovery by Takahashi and Vodyanik, iPSCs generated from patient's somatic cells have opened up a new avenue to exploring the mechanisms of PD and developing the stem cell-based personalized therapeutic strategy.

## 3. iPSCs Models for PD

Over the years, iPSCs derived from the patients and then differentiated into disease-relevant cell types have been widely used to mimic the phenotype of diseases and have achieved great progress in pathogenic mechanism studies. In terms of nervous system disorders, patient-derived iPSCs have been used to differentiate into motor neurons, astrocytes, DA neurons, or other cell types of the affected diseases. iPSCs generated from Alzheimer's disease (AD), Amyotrophic Lateral Sclerosis (ALS), spinal muscular atrophy (SMA), and PD patients with genetic mutations were successfully differentiated into neuronal cells, and disease-related pathologic phenotypes have been identified in these cells. Furthermore, patient iPSC-derived neuronal cells offer a direct insight into the early-stage and progressive pathologic alternations in disease, further recapitulating the molecular pathogenesis of diseases [11–14].

Since PD is a kind of disease with progressive degeneration of dopaminergic (DA) neurons, iPSC differentiated DA neurons seem to be the appropriate model to decipher physiological and pathological mechanisms of PD. As is described above, iPSCs can be generated by specific transcription factors, viral vector, or other small molecules and are then differentiated into DA neurons. The methods of DA neurons differentiation include targeted differentiation and direct lineage conversion. Targeted differentiation strategy is similar to embryonic stem cell (ESC) induction, which has been widely used to differentiate DA neurons, and the protocol has been constantly modified or refined. In 2009, Chambers and colleagues demonstrated that, with the combination of Noggin and SB431542, two inhibitors of SMAD signaling in differentiation and the efficiency of iPSC differentiation could be improved, which allowed for the complete induction into neuronal cells [15]. The second strategy for obtaining DA neurons is the direct lineage conversion. Somatic cells can be first induced into neural precursor cells (iNPCs), which are then differentiated into astrocytes and DA neurons. More importantly, as the generation of iNPCs is a gradual process, the donor transcription factors are silenced over time, which lowers the risk of tumors. Direct lineage conversion provides a new source of human cells for stem cell based replacement therapy and holds promise for application in drug discovery and screening [16, 17].

In 2009, Soldner et al. generated iPSCs from sporadic PD patients by using modified lentiviruses carrying loxP sites flanking the integrated provirus for the first time. This strategy not only improved the efficiency of reprogramming but also allowed the removal of the transgene sequences to generate iPSCs free of reprogramming factors [14]. Over the past few years, somatic cells from patients with* SNCA*,* LRRK2*,* PINK1*, and* Parkin* mutations and at-risk individuals carrying* GBA* mutations have been successfully induced to iPSCs and differentiated into DA neurons. With the help of these techniques, the pathogenesis of PD has become clearer.

### 3.1. *SNCA*

*SNCA* encodes *α*-synuclein protein. Missense mutations (such as A53T, E46K, and A30P) and genomic multiplications (duplication and triplication) of* SNCA* were reported in many autosomal dominant PD patients. Patients with* SNCA* mutations were characterized by a loss of DA neurons in SNpc and *α*-synuclein accumulation in neurons—the pathological hallmark of PD [8]. However, the definitive pathogenic mechanism caused by* SNCA* mutations is still elusive, although it is widely believed that *α*-synuclein aggregation and cellular toxicity may contribute to the course of neuronal degeneration [18, 19].

In 2011, Soldner et al. generated iPSCs from two early onset PD patients with A53T and E46K mutations by combining zinc-finger nuclease- (ZFN-) mediated genome editing and iPSC technology, but the phenotypic changes of these cells were not reported [20]. The accumulation of *α*-synuclein in DA neurons is a common pathological change in PD patients, which also exist in iPSC generated DA neurons derived from patients with* SNCA* triplication. In 2011, Devine and Byers reported that PD patients with *α*-synuclein triplication (AST) and unaffected controls showed no difference in ectopic expression of *α*-synuclein. When differentiated into DA neurons, the quantity of *α*-synuclein was doubled in AST neurons compared with neurons from the controls. Furthermore, AST neurons were more sensitive to peroxide induced oxidative stress, further substantiating the role of *α*-synuclein accumulation and oxidative stress in PD [21, 22]. Also, their findings were consistent with a previous study in blood and brain tissue from the patient with* SNCA* triplication, which showed higher levels of *α*-synuclein compared with controls [23].

### 3.2. *LRRK2*

*LRRK2* is a member of the leucine-rich repeat kinase family, encoding a protein that has GTPase and kinase functions. The dysfunction of* LRRK2* was reported to be associated with impaired dendritic neuronal arborization and autophagy [24, 25].* LRRK2* mutations are the most common cause of familial PD. Mutations including N1437H, R1441C, and G2019S of* LRRK2* were reported in autosomal dominant PD patients, and G2019S is by far the most common mutation, especially prevalent among Ashkenazi Jews [8, 26].

iPSCs derived from PD patients with G2019S mutation were generated in many groups. Oxidative stress, *α*-synuclein accumulation, autophagy, and damaged mitochondrial DNA were reported in these iPSC-derived DA neurons and the pathogenesis of PD was further investigated. Compared with unaffected DA neurons, the G2019S-iPSC-derived DA neurons were more sensitive to oxidative stress (such as hydrogen peroxide, MG-132) or proteasomal stress-induced apoptosis, and they also exhibited an increased *α*-synuclein protein level after long-term cultivation [27, 28]. In addition to its role in oxidative stress and *α*-synuclein protein accumulation, impaired autophagic clearance and morphological alterations (including reduced numbers of neurites and neurite arborization) can be seen in these DA neurons [29]. More importantly, another study showed damaged mitochondrial DNA (mtDNA) in G2019S-iPSC-derived DA neurons, and the damage can be reversed by zinc finger nuclease-mediated repair. It suggested that mtDNA damage might be induced by* LRRK2* mutations, and oxidative stress, *α*-synuclein accumulation, and impaired autophagy together with damaged mtDNA may play interactive roles in the course of PD [30].

### 3.3. *PINK1*

*PINK1* encodes a mitochondria-targeted kinase, which can protect neuronal cells from stress-induced mitochondrial dysfunction [8]. In 2013, Rakovic et al. demonstrated that iPSCs derived from* PINK1* mutation carriers showed a deficiency of endogenous* Parkin* levels to initiate mitophagy upon loss of the mitochondrial membrane potential due to ubiquitination dysfunction, suggesting that* PINK1* may play an overlapping role with* Parkin* in mitophagy [31]. In 2011, Seibler et al. reported that mutant* PINK1* iPSC-derived DA neurons showed impaired recruitment of* Parkin* to mitochondria upon depolarization, increased mitochondrial copy number, and upregulation of PGC-1*α*. More importantly, another study reported that lentiviral overexpression of wild type* PINK1* in these DA neurons was able to restore the translocation of* Parkin* to mitochondria, further validating the association between* PINK1* and* Parkin* [32]. In addition, these cells showed a decreased mitochondrial membrane potential and mitochondrial complex I activity, which was consistent with the results of previous studies from other groups [33, 34]. Therefore, Seibler and Rakovic generated a valuable cellular model closely resembling the phenotype reported in PD patients and highlighted the importance of* PINK1* mutation-caused mitochondrial dysfunction in pathogenesis for PD.

### 3.4. *Parkin*

*Parkin* encodes a component of the E3 ubiquitin-ligase complex, which mediates the targeting of substrate proteins for proteasomal degradation. Besides a concerted role with PINK1 in mitophagy and oxidative stress,* Parkin* is also associated with dopamine homoeostasis. iPSC-derived DA neurons from patients with* Parkin* mutation showed decreased DA uptake and increased spontaneous DA release. There was also an increased level of reactive oxygen species (ROS) in these neurons as a result of mitochondrial dysfunction, which indicates that* Parkin* can enhance the precision of DA neurotransmission and suppress the oxidation of DA [35, 36]. Furthermore, these cells exhibited similar pathological changes seen in G2019S-iPSC-derived neurons, including the accumulation of *α*-synuclein and its correlation with Lewy body formation [35].

In addition, mutant* Parkin* iPSC-derived DA neurons showed a reduced neurite length and complexity due to destabilization of microtubules, which could be rescued by overexpressing wild type* Parkin* in these neurons. Their findings supported that microtubule stabilization maintains the morphological complexity in neurons, and the dysfunction of* Parkin* damages not only the morphology of DA neurons but also neuron survival [37].

### 3.5. *GBA*

*GBA* encodes a lysosomal membrane protein *β*-glucocerebrosidase (also known as acid *β*-glucosidase), the mutation of which results in accumulation of glycolipid substrates in lysosomes, leading to an autosomal recessive lysosomal storage disorder—Gaucher disease [38].

Mutations in* GBA* were thought to be a risk factor for PD in different ethnic groups [39, 40]. Glucocerebrosidase deficiency and lysosomal dysfunction were thought to be an important pathogenic mechanism for PD [41]. In 2012, Panicker et al. reported that iPSC-derived DA neurons from patients with Gaucher disease showed a high level of *α*-synuclein protein and decreased clearance ability in macrophages due to glucocerebrosidase (GCase) deficiency. On the contrary, the overexpression of *α*-synuclein inhibits the intracellular trafficking of GCase, which can decrease the activity of lysosomal GCase [42, 43]. The findings suggested a bidirectional effect between a-synuclein accumulation and GCase deficiency, further supporting the important role of *α*-synuclein neurotoxicity and autophagy-lysosomal pathways in the process of PD occurrence.

Though iPSCs derived from patients with causative or at-risk mutations have successfully modeled PD and further illustrated the pathogenic pathway of the disease, the pathogenesis of neurodegeneration in PD remains elusive. Further studies of iPSC-derived DA neurons from patients with other genetic mutations (such as* VPS35*,* DJ-1*, and* PLA2G6*) are needed to model PD and elucidate the pathogenesis.

## 4. Potential Clinical Applications of iPSCs

Taking all described above together, patient-derived iPSCs seem to be an ideal model to recapitulate the disease-related phenotypes and the pathological changes of diseases, as these cells are able to differentiate into any cell types of human body for disease modeling and mechanism exploring. Indeed, iPSCs have served as potential cell tools for clinical applications, some of which even achieved promising results. iPSCs derived from PD patients were applied for drug discovery, replacement therapy, or other biological applications, aiming at realizing personalized treatment and transforming biomedical research into clinical application.

### 4.1. iPSCs Models in Drug Discovery

In 2012, Cooper et al. generated DA neurons from individuals carrying PINK1 Q456X, LRRK2 G2019S, and R1441C mutations. They found that these cells were more vulnerable to PD associated chemical toxins valinomycin and concanamycin A. Moreover, the mutant PINK1 iPSC-derived neurons showed increased mitochondrial reactive oxygen species (mROS) concentrations when a low concentration of valinomycin was added. Accompanied with the increased mROS in these neurons, the level of glutathione (GSH), an important antioxidant to prevent damage caused by mROS concentrations, was decreased. Importantly, damage induced by these chemical toxins could be rescued by coenzyme Q10, rapamycin, and GW5074 (a kind of LRRK2 kinase inhibitor). Their results suggested that iPSC-derived cells are an ideal model for pharmacological study, and that coenzyme Q10, rapamycin, and GW5074 may save the damaged DA neurons and prevent them from progressive degeneration [44, 45].

### 4.2. iPSCs for Cell Replacement Therapy

Stem cell based therapy for PD can be traced back to three decades ago; from then on, scientists have been striving to advance the therapy and have got varying results for that. In 2008, Mendez et al. reported that PD patients who had DA neurons implanted from fetal midbrain cell suspensions lived 14 years without pathology. Recently, another group reported long-term clinical outcomes for fetal mesencephalic tissue (rich in dopaminergic neuroblasts) transplantation in two PD patients, showing an improvement of their motor symptoms free of any pharmacological dopaminergic therapy. Their findings proved that DA neurons transplantation might offer a long-term symptomatic relief in PD patients [46, 47]. On the other hand, some significant side effects, such as graft-induced dyskinesia and dystonia, occurred in patients who received fetal nigral transplantation [48, 49]. Considering these adverse effects, the use of stem cell-derived DA neuron transplantation in PD patients remains controversial.

After Takahashi et al. and Vodyanik et al. induced adult human somatic cells into iPSCs with a series of transcription factors (Oct4, Sox2, Klf4, c-Myc or Oct4, Sox2, Nanog, and Lin28), the transplantation of DA neurons for PD patients had become more feasible and easily operable. iPSCs can be obtained from human somatic cells, which avoids the ethical problems of applying human embryos for study. However, the risk of tumorigenicity and other unpredictable adverse effects were raised due to viral vector insertions and c-Myc oncogene reactivation [50].

In recent years, many studies have reported that transplanted iPSC-derived neurons were able to increase regeneration and functional recovery in ischemic stroke rat model [51, 52]. Notably, an improvement in motor ability was found in a PD rat model after the transplantation of human derived induced neural stem cells (iNSCs) into the striatum of the rats. In addition, in vivo study proved that these iNSCs were able to survive and differentiate into DA neurons, suggesting that iPSC-derived neuron transplantation can replace the lost neuronal cells and rescue the damaged function of neurons [53, 54]. Putting the adverse effects aside, patients' somatic cell-derived iPSC transplantation may be a potential personalized cell strategy to treat PD or other degenerative diseases in future with little or no immune reaction.

## 5. Challenges and Future Directions

As described above, iPSCs derived from patients with different genetic mutations or carrying at-risk mutations are an ideal model for studying the pathophysiological mechanisms underlying PD. Its potential applications in drug discovery and cell replacement therapy, will support an improved life quality of the patients.

Despite the fact that iPSC technology is still ongoing and has been greatly improved to accelerate the development of clinical trials, there still exist several challenges and limitations in iPSC transplantation for PD. Firstly, iPSCs are induced by viral vector insertion of transcription factors, which is accompanied with tumorigenicity or other adverse effects. Safer and more effective transduction methods have been attempted in many studies, successful application of these new methods will not only save time but also improve reprogramming efficiency and safety [3–7]. Until now, there is no study reported using iPSC-derived neurons for transplantation in PD patient. Secondly, because our human is an integrated complex system, different cell types can play an interactive role with each other. Though iPSCs can model PD, it would be difficult for these cells to reveal the exact pathophysiology status of human. Last but not least, as PD is a neurodegenerative disease, whether the transplanted neurons will function as expected for long term is still unknown. Moreover, ethical issues before transplantation should also be taken into consideration [55]. Taken together, further investigations are required for iPSC-derived DA neuron transplantation in rodents and nonhuman primates to evaluate the long-term clinical benefits and potential adverse effects. The road toward clinical application of iPSC-based therapy is promising, but we still have a long way to go.
